# Guilt, shame, and embarrassment: similar or different emotions? A comparison between Italians and Americans

**DOI:** 10.3389/fpsyg.2023.1260396

**Published:** 2023-12-12

**Authors:** Cinzia Giorgetta, Francesca Strappini, Alessandra Capuozzo, Elisa Evangelista, Antonella Magno, Cristiano Castelfranchi, Francesco Mancini

**Affiliations:** ^1^Schools of Cognitive Psychotherapy (APC-SPC), Rome, Italy; ^2^Department of Psychology and Cognitive Sciences, DipSCo, University of Trento, Rovereto, Italy; ^3^Department of Philosophy and Communication, University of Bologna, Bologna, Italy; ^4^Neuromotor and Cognitive Rehabilitation Research Center, Section of Physical and Rehabilitation Medicine, Department of Neuroscience, Biomedicine and Movement Sciences, University of Verona, Verona, Italy; ^5^Institute of Cognitive Sciences and Technologies, National Research Council of Italy, Rome, Italy; ^6^Department of Human Sciences, Università Degli Studi Guglielmo Marconi, Rome, Italy

**Keywords:** emotions, cultural diversity, guilty, ashamed, embarrassed, vignette

## Abstract

**Introduction:**

Guilt, shame, and embarrassment represent affective experiences with social implications and diverse self-relevant negative affect. While the distinction between these emotion terms has been extensively investigated, little is known about how they diverge and are related to each other and their crosscultural differences.

**Methods:**

Here, we used a community sample (*N* = 163) comprised of Americans and Italians and a scenario-based measure in which we asked participants to report the intensity of emotions that the story’s main character would feel. The elements used to build the scenarios were based on a recent theoretical approach that proposes distinguishing cognitive, somatic, interoceptive, and behavioral ingredients to differentiate between these emotions. We hypothesized that these ingredients might effectively elicit the target emotions and that the main differences across these cultures would be associated with the emotion terms of shame/vergogna.

**Results:**

Our findings suggest that these defining elements are effective in evoking experiences of guilt, shame, and embarrassment. Moreover, we found that shame was equally elicited by the Shame and Guilt Scenarios only in the American sample, thus suggesting a proximity between shame and guilt in the American sample compared to the Italian’s terms of vergogna and colpa.

**Discussion:**

These results suggest important implications for the psychology of moral emotions and highlight the importance of taking into account some cognitive factors, such as the quality of self-evaluation, the discrepancy between the actual self and the ideal self vs. the sense of perceived responsibility, and the different domains related to self-esteem.

## Introduction

In our daily life, we often tend to use the terms embarrassment, shame, and guilt as synonyms, as these emotions labels are often confused and used interchangeably. The source of this confusion might originate from several factors as these emotions are phenomenologically interrelated, they often coexist (Harder, 1995; Ferguson and Crowley, 1997), their neural correlates partially overlap (Bastin et al., 2016; Piretti et al., 2023), and they might not be precisely defined in the common language (Tangney et al., 1996). Moreover, although a large body of research considers these terms distinct emotions, the scientific debate about the characteristic features is still open. Furthermore, there is a growing recognition that the exact labels used to indicate emotions can vary in their meaning and experience across different cultures and languages and that emotions, culture, and language influence each other and are closely intertwined (Jack et al., 2012; Mesquita et al., 2015; Heyes, 2019; Dylman et al., 2020). Indeed, numerous studies highlighted the difficulties in translating the words that label the emotions from one language to another one (Brislin, 1970; Wierzbicka, 1986, 1992, 1994, 1997; Heider, 1991; Russell, 1991; Mesquita and Frijda, 1992; Russell and Sato, 1995; Mesquita, 2001; Parkinson et al., 2005; Giorgetta et al., 2012).

Most cross-cultural taxonomic studies have proposed that shame is one of the most problematic emotion labels (Mauro et al., 1992; Storm and Storm, 1992; Fontaine et al., 2002) and that some of its translations have been located in clusters unrelated to shame (Shaver et al., 1992; Church et al., 1998; Kobayashi et al., 2003). Indeed, much empirical evidence seems to suggest that the translations of shame are not always linked to the same cluster of conceptual features and that cannot be considered as an equivalent term (e.g., Menon and Shweder, 1994; Hurtado de Mendoza et al., 2010). For instance, some authors have found that the Spanish word verguenza includes the concept of shame and embarrassment, but the verbal category shame also overlaps with the Spanish term culpa (guilt; Iglesias, 1996; Hurtado de Mendoza and Parrott, 2002; Pascual et al., 2007). An influential review of anthropological studies found that shame does not have an exact equivalent in many cultures, such as Japan, Indonesia, Nepal, Tahiti, and Aboriginal Australia (Russell, 1991).

Certainly, shame, guilt, and embarrassment are considered, at varying degrees, unpleasant experiences and are generally considered to be self-conscious, moral, or social emotions as they are related to the sense of self and awareness of the reactions of others toward us (Barrett, 1995; Lewis, 1995; Tangney and Fisher, 1995). Moreover, they involve negative self-evaluations and feelings of distress caused by the perception of having made mistakes/failures or transgressions (Tangney et al., 2007). Guilt implies a negative moral self-evaluation where morality refers to behaviors, goals, beliefs, or traits taken into account. Shame instead implies a negative “non-moral” self-evaluation where “non-moral” refers to the fact that it is not focused on accountability issues. Guilt comes out from the transgression of own moral standard, when the moral value is compromised, and the person is responsible for being able to harm because of his/her action or omission, whereas shame arises when the ideal self and the purpose of the good image are compromised (Sabini and Silver, 1997; Rozin et al., 1999; Miceli and Castelfranchi, 2018).

Some authors consider guilt as a “moral and prosocial” emotion, while shame is considered an “ugly and anti-social” emotion (Tangney and Tracy, 2012). Based on their physiological expressions, guilt is characterized by a sense of oppression in the chest, while shame is characterized by redness, bowing the head, and lowering the gaze (Miceli and Castelfranchi, 2018). Regarding the tendency to action, guilt refers to the desire to apologize, be forgiven, or repair the damage, while shame refers to a desire to sink, disappear, and be elsewhere (Tangney and Dearing, 2002; Miceli and Castelfranchi, 2018). Guilt is more connected to private episodes than shame, while shame is more related to public episodes than guilt. Indeed, according to Smith et al. (2002), guilt is associated with events with moral value and with “remorse,” “regret,” and troubled consciousness, while shame is associated with the sense of inferiority regardless of whether it is public or private, and it is linked to “humiliation” and “embarrassment.” Several studies showed that although shame is experienced in public much more than guilt, it can also be experienced in private if one thinks it could go public or self-evaluate (Lewis, 1997, 2008). Guilt is also more associated than shame with the violation of internal standards (Smith et al., 2002). According to several authors (e.g., Sabini and Silver, 1997; Smith et al., 2002), guilt is elicited by moral transgressions and implies errors for which one feels responsible, while shame includes particularly non-moral errors, i.e., issues attributable to physical defects, incompetence, inadequacy for which the person is not responsible.

Several studies have suggested crucial distinctions between these emotions accounting for differences in their cognitive, behavioral, and somatic outcomes, and have investigated their specificity interrelation (Sabini et al., 2001; Tangney et al., 2005; Lickel et al., 2011; Miceli and Castelfranchi, 2018). However, although shame and guilt have received considerable attention in the literature (e.g., Lewis, 1971; Tangney, 1993; Sabini and Silver, 1997; Tangney, 1999; Tangney and Dearing, 2002; Miceli and Castelfranchi, 2018), less attention has been paid to embarrassment (Tangney, 1993; Lewis, 1995; Keltner and Buswell, 1996), and only a few studies have focused on the differences between shame, guilt, and embarrassment.

According to the existing literature, embarrassment is considered an emotion quite distinct from shame and guilt and significantly different across the affective, cognitive, and motivation dimensions (Tangney et al., 1996). In particular, it has been shown that people experiencing shame or guilt feel more responsible, regretful, disgusted, and angry toward themselves than people experiencing embarrassment (Tangney et al., 1996). They also perceive that others feel more disgusted and angry toward themselves than when they feel embarrassed. In contrast, embarrassment arose from trivial and humorous events and occurred more suddenly and with a greater sense of surprise. It is accompanied by more visible physiological changes (e.g., blushing, increased heart rate) and a greater sense of exposure and conspicuousness (Tangney et al., 1996). As for the emotion of shame, embarrassment is characterized by being an internal state, even though it is experienced at a lower intensity level (Lewis, 1971; Tomkins, 1987; Borg et al., 1988; Kaufman, 1989; Lewis, 1992). It has been proposed that embarrassment arises from the violation of conventions (Keltner and Buswell, 1996; Tangney et al., 1996), and it is associated with both real and apparent defects as opposed to shame, which is instead associated with only real defects (Sabini et al., 2001). On the other hand, some studies have questioned that embarrassment (compared to shame or guilt) results from more considerable losses of perceived approval from others than from changes in self-appraisal (Buss, 1980). In embarrassment, shame, and guilt, people evaluate themselves more harshly than they believe others do. Indeed, embarrassed people typically believe they have made more negative impressions on others than they actually did (Semin, 1982). Embarrassment is supposed to focus more on one’s self-presentation than self-evaluation, as it is for shame (Klass, 1990). Finally, while in shame, there is no conflict of choice, in embarrassment, there is a conflict of choice associated with uncertainty regarding a decision that can potentially compromise one’s self-image (Castelfranchi and Poggi, 1990).

Given the relevant role that these complex emotions have not only within experimental psychology and psychopathology research but also the clinical practice (Shafran, 1997; Mancini and Gangemi, 2004, 2018; D'Olimpio et al., 2013; Perdighe et al., 2015; Melli et al., 2016), it is crucial to know which are the distinctive elements that differentiate them. In order to answer this question, here we used a scenario-based approach based on the distinguishing criteria for guilt, shame, and embarrassment proposed by Castelfranchi et al. (1989), Castelfranchi (1998) and Castelfranchi and Poggi (1990). The scenarios were built based on the criteria domains proposed by the authors: the type of self-evaluation involved (inadequacy vs. harmfulness); the focus on the perceived discrepancy between the actual self and the ideal self vs. the focus on the sense of perceived responsibility for someone’s harm; and the involvement of the different domains related to self-esteem (Miceli and Castelfranchi, 2018). Specifically, for each emotion label, we defined some prototypical elements to determine and differentiate the three emotions across the scenario types. These elements were created using cognitive factors, tendency to action and interoceptive/somatic factors that, according to Miceli and Castelfranchi (2018), Castelfranchi (1998), and Castelfranchi and Poggi (1990), are considered fundamental elements to define these emotions, as described in Table 1. Next, we compared the emotion ratings associated with each scenario type across two different cultural contexts, Italian and American, which are defined, respectively, as more collectivist the first than the second (e.g., Burton et al., 2021). We hypothesized that each scenario type would primarily elicit the emotion target consistently with the specific elements employed. In particular, we conjectured that: feeling responsible for the actions, thinking that there is a victim caused by an unjust damage, self-criticism, a tendency to take action to repair the arm, and feeling tightness in the chest and restless, would be mainly associated with guilt; thinking that there is a shared value, a damage in the self-image, a desire to disappear and look down, and feeling blushing and hot flashes would be mainly linked with shame; thinking that the situation is uncertain and unclear, feeling not knowing what to do, having doubts on the correct actions to take, thinking that the self-image might be compromised (in presence of known or unknown people) would mainly elicit embarrassment (Table 1).

**Table 1:** Prototypical elements used to create the different scenarios.

	Scenarios			
Elements referred to the protagonist of the story	Guilt	Shame	Embarrassment1	Embarrassment2
Feeling an emotional experience	×	×	×	×
Feeling responsible for the actions	×	/	/	/
Thinking that there is a victim (unjust damage)	×	/	/	/
Self-criticism	×	/	/	/
Tendency to take action to repair the harm	×	/	/	/
Feeling tightness in the chest and restlessness	×	/	/	/
Thinking that there is a shared value	/	×	/	/
Thinking that there is a damage in the self-image	/	×	/	/
Desire to disappear	/	×	/	/
Looking down	/	×	/	/
Feeling blushing and hot flashes	/	×	/	/
Thinking that the situation is uncertain and unclear	/	/	×	×
Feeling not knowing what to do	/	/	×	×
Doubts on the correct action	/	/	×	×
Thinking that the self-image might be damaged	/	/	×	×
Thinking that the self-image might be damaged in presence of familiar, known people	/	/	×	/
Thinking that the self-image might be damaged in presence of unknown people	/	/	/	×

Cognitive, somatic, interoceptive, and behavioral ingredients used to define the scenarios.

According to the existing theory and findings related to the cross-cultural studies that compared the more collectivist cultures of East Asia (e.g., China and Japan) and Europe (e.g., Spain) versus the more individualistic culture of the American (e.g., Wong and Tsai, 2007; de Hurtado de Mendoza et al., 2010; Krawczak, 2014) we expected that the main differences between the American and the Italian culture would be in relation to the emotion labels of shame/vergogna. Specifically, we hypothesized that the scenario inducing guilt would elicit more shame in the American than in the Italian sample, in line with an Anglo-Saxon conception of shame caused by moral transgressions and characterized by negative internal, global, and stable attributions (Niedenthal et al., 1994; Lewis, 2000; Tangney and Dearing, 2002; Tracy and Robins, 2004; Hurtado de Mendoza et al., 2010). Indeed, shame is usually elicited in Americans in violation of a moral standard, in relation to events involving a sense of responsibility for what happened, and linked to making amends (Miller and Tangney, 1994; Tangney and Fisher, 1995; Keltner and Buswell, 1996; Tangney et al., 1996). From this perspective, we further hypothesized that shame would be closer to guilt than embarrassment, only in the American sample (Tangney et al., 1996).

## Methods

### Transparency and openness

We report how we determined our sample size, all data exclusions, all manipulations, and all measures in the study, and we follow JARKS (Kazak, 2018). All materials have been made publicly available at the Open Science Framework (OFS) and can be accessed.^1^ Data were analyzed with Jamovi 2 (Selker, 2017; see below for more details). This study’s design and its analyses were not pre-registered.

### Participants

Participants included 206 individuals recruited online across Italy and the American. One-hundred-eight participants ranging from 20 to 50 years of age (56 females; M_age_ = 34.54 years, SD = 7.46; education M_age_ = 16.18 years, SD = 4.08) were enrolled for the Italian sample. For this group, we recruited only participants with Italian nationality and native Italian speakers. For the American sample, we recruited 80 participants ranging from 20 to 50 years of age (43 females, two other; M_age_ = 34.85 years, SD = 7.60; education M_age_ = 16.52 years, SD = 2.14). For this group, we enrolled only participants with American nationality and Native American English speakers. Nationality was classified according to participant’s responses to a standard demographic question.

Although no prior study has examined this specific research question, a previous study investigating the differences in participants’ ratings of shame and embarrassment emotions found a moderate effect size (study 1; Sabini et al., 2001). According to the effect size (Cohen’s *d* = 0.5), 67 participants should be sufficient to find a significant difference between ratings across conditions, considering a power of 98% and a level of significance of 5% (two-sided; G*Power 3.1; Faul et al., 2007). Thus, our samples are adequate for the study’s primary objective.

Data were collected from September 2020 through March 2021. Participants gave written informed consent, were paid for their time, and were naïve to the purpose of the experiments. Written informed consent and data collection were performed using a web-based interface through Google Forms^2^ for the Italian sample and Testable (accessed on 1 March 2021)^3^ for the American sample.

The work was carried out in accordance with the Code of Ethics of the World Medical Association (World medical association, 1964) and was approved by the Ethics Committee of the Association of Cognitive Psychotherapy (APC-SPC; Prot. N. 8–2023).

### Procedure and materials

We used the elements described by Castelfranchi (1998) and Castelfranchi and Poggi (1990) to create four types of scenarios (see Table 1). Each scenario was designed to evoke primarily one of three emotions: guilt/colpa, shame/vergogna, and embarrassment/imbarazzo. However, we designed the scenarios such that some sentences may evoke more than one emotion. The scenarios and the questions were presented to the two groups in the native language.

The task consisted of 16 scenarios, of which four were primarily guilt-emotion scenarios, four were shame-emotion scenarios, and eight were embarrassment-emotion scenarios. We defined two types of embarrassment scenarios to disentangle the effect of the context (being in the presence of known or unknown people, Embarassment-1 and -2, respectively). The elements used to differentiate each scenario type are described in Table 1.

Participants were asked to read the scenarios presented randomly and rate the subsequent questions. In performing the task, we expressly ask participants to take into account solely and exclusively the information explicitly reported in the story. Each questionnaire consisted of nine items and asked subjects to imagine themselves in each of the scenarios and to rate the emotions the main character would feel. Beneath each scenario were 10-point rating scales for six emotions, randomly presented: guilt/colpa, shame/vergogna, embarrassment/imbarazzo, anger/rabbia, fear/paura, and regret/rammarico. The scales ranged from 1 (not at all) to 10 (extremely). Our primary focus was on the first three emotions; the others were included partly to disguise our purpose.

We included three additional scales, happiness, responsibility, and reality, to assess the validity of subjects’ ratings. The happiness scale, which ranged from −5 (very unhappy) to 5 (very happy), was presented as the first question and assessed the negative valence of the scenarios. The responsibility (ranging from 1 to 10) assessed how much responsibility the main character would feel for the actions, and it was also a relevant factor to consider in relation to guilt. Finally, participants rated how realistic the scenario was on a scale from 1 (not at all) to 10 (extremely). Examples of scenarios are given in the Appendix/SI.

The entire session lasted about 40 min.

### Psychometrics

At the end of the experiment, participants completed a computerized version of the Toronto Alexithymia Scale (TAS-20; Bagby et al., 1994) to assess the alexithymia. For the Italian sample, we used an Italian version validated by Bressi et al. (1996). The questionnaire TAS-20 scores range from 20 (no alexithymia) to 100 (alexithymia present). Only participants with a TAS-20 score lower than 61 were included in the study.

### Scenarios design

The scenarios were built based on the criteria domains proposed by Castelfranchi et al.: the type of self-evaluation involved, such as feeling guilt or self-criticism linked to the inadequate behavior; the focus on the perceived discrepancy between the actual self and the ideal self vs. the focus on the sense of perceived responsibility for someone’s harm; and the involvement of the different domains related to self-esteem. Indeed, according to Miceli and Castelfranchi (2018), shame and embarrassment focus more on the desired self-imagine, while guilt focuses more on the given damage, as shame seems to be more involved in low self-esteem compared to guilt, probably because shame is strongly connected to the risk to have a bad self-image, while guilt is strongly connected to the given damage and so on the subsequent action to repair (Miceli and Castelfranchi, 2018).

### Statistical analyses

**Figure 1:**
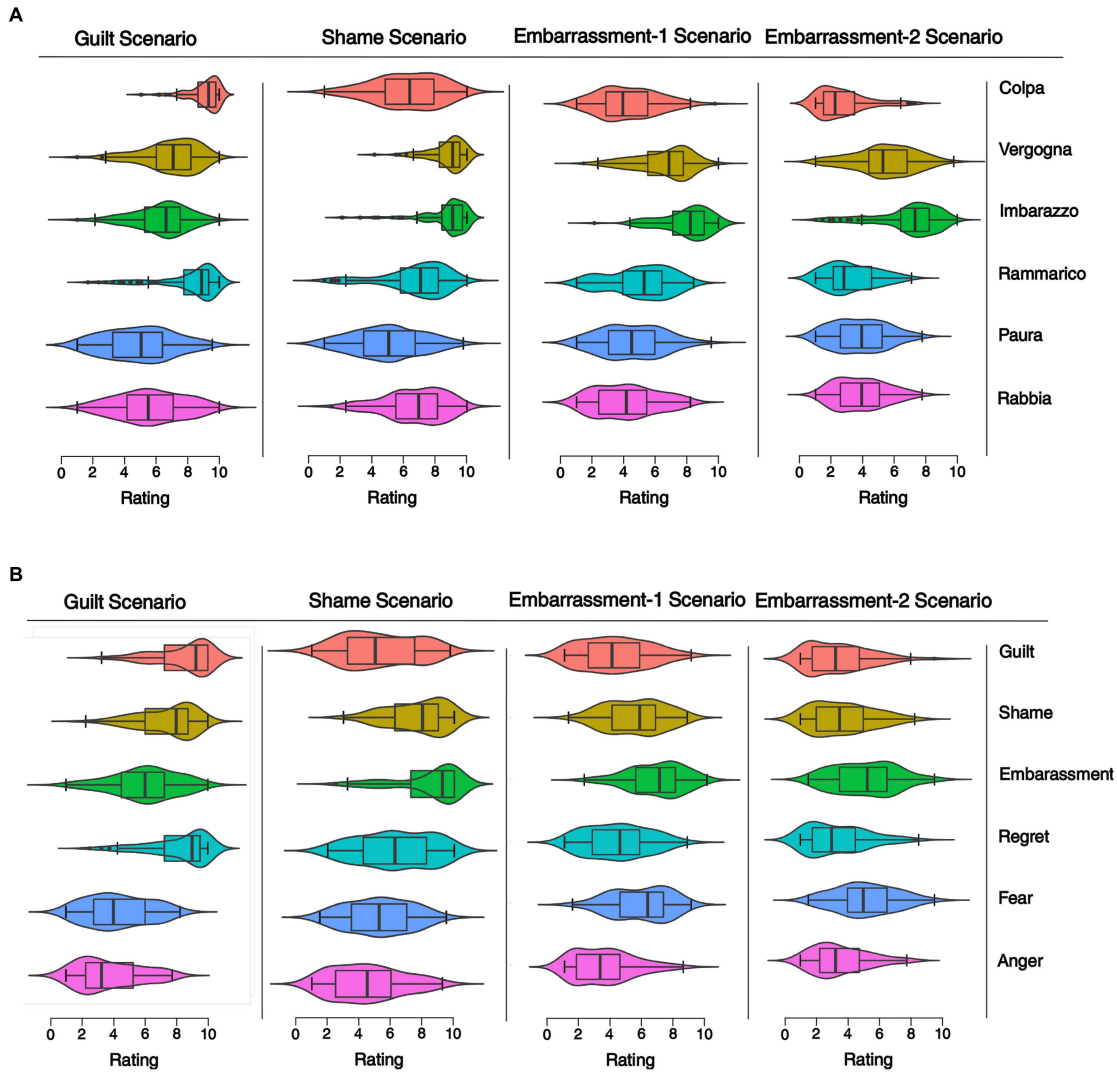
Emotions rating across scenarios in the Italian and American samples. The figure shows the violin plots with the superimposed box plots of the self-reported emotions intensity across the four scenarios (shame, guilt, embarrassment-1, embarrassment-2) in the Italian (panel **A**) and American groups (panel **B**). The violin plots show the distribution of ratings for each scenario. The box plots show the median rating (the dark horizontal lines), the 75th percentile to the 25th percentile (the boxes), and the upper and lower boundaries 1.5*75th (or 25th) quartiles (the whiskers). Ratings were provided on a scale from 1 (*not at all*) to 10 (*extremely*) in response to the question “How strongly the main character feels this emotion?”.

Descriptive statistics were computed for each scale and presented as mean, median, and standard deviation (Figure 1).

The internal consistency of each scale was determined by the McDonald’s omega (ω) coefficient, following Hayes and Coutts (2020) recommendation, independently for the two groups. The coefficient was computed utilizing the “Factor” package in Jamovi 2 (Selker, 2017; Caldwell, 2022; The Jamovi project, 2021; R Core Team, 2021), which is part of the “lavaan” package for R (Rossel, 2012).

First, we computed two repeated-measures ANOVA, one for each group, with the within-subject factors Scenario (Shame, Guilt, Embarrassment-1, Embarassment-2) and Emotion (guilt/colpa, shame/vergogna, embarrassment/imbarazzo, anger/rabbia, fear/paura, regret/rammarico), and the factor Sex (female, male) to assess possible gender effects.

Although there are many ways to analyze the resulting emotion ratings, our choices were guided by a desire to address our two main questions.

To investigate possible differences in the average of the emotions ratings between Italians and Americans, we performed a series of mixed ANOVAs. First, we performed three ANOVAs, one for each *control scale* (happiness, responsibility, and reality), to compare participants’ rating validity across Scenarios (Shame, Guilt, Embarrassment-1, Embarrassment-2; within-subjects factor) and Nationality (Italian, American; between-subjects factor). Next, we tested whether the average of the emotions ratings differed between Italians and Americans in each scenario type. To that end, we conducted four ANOVAs, one for each scenario (Shame, Guilt, Embarrassment-1, Embarrassment-2), considering Emotion (shame, guilt, embarrassment, regret, fear, and anger; within-subjects) and Nationality (Italian, American; between-subjects) as factors. Finally, we performed three ANOVA, one for each emotion, with the within-subjects factor Scenario and the between-subjects factor Nationality to compare the two groups in how each target emotion was rated across scenarios.

Lastly, chi-squared tests were used to examine differences in the rating frequencies among the two groups on Shame and Guilt scenarios.

Violations of the sphericity assumption in the mixed ANOVA omnibus tests were corrected using the method proposed by Greenhouse and Geisser (Greenhouse and Geisser, 1959). Violations of normality and homoscedasticity were assessed with a visual inspection of the normal quantile-quantile plots of the standardized residuals and with the homogeneity of variance test (Levene’s test, Levene, 1960). Analysis of variance is considered reasonably robust to possible violations when the size of groups is reasonably similar (e.g., largest/smallest = 1.5); thus, our sample ratio of 1.5 is considered adequate (Stevens, 1996, p. 249; Blanca Mena et al., 2017, 2023).

All the analyses were performed in jamovi (the jamovi project, 2022; version 2.3; Computer Software; retrieved).^4^ The ANOVAs were performed utilizing the “afex” package (Singmann et al., 2015), and the level of statistical significance was set at α = 0.05. Post-doc comparisons were Bonferroni corrected for multiple comparisons.

## Results

### Overview

In the Italian group, 10 subjects were excluded due to the presence of alexithymia, leaving a total sample of 98 participants ranging from 22 to 50 years of age (56 females, M_age_ = 34.36 years, SD = 7.48; education M = 16.45 years, SD = 2.84).

In the American group, 13 subjects were excluded due to the presence of alexithymia, leaving to a total sample of 65 participants ranging from 20 to 50 years of age (32 females, one other; M_age_ = 34.66 years, SD = 7.53; education M = 14.45 years, SD = 2.07).

We created the total average scores for all rated emotions and the three control scales (happiness, responsibility, and reality) in the Shame, Guilt, Embarrassment-1, and − 2 scenarios. All the analyses were performed using these averaged scores (see Figure 1).

Preliminary analyses revealed no significant main effects or interactions involving gender, and therefore gender was not included in the following analyses (Italian: F_6.09, 584.791_ = 1.667, *p* = 0.126, ηp^2^ = 0.017; American: F_4.71,367.59_ = 1.30, *p* = 0.192, ηp^2^ = 0.016).

#### Manipulation check

McDonald’s omega coefficients were computed for the three target emotions across the scenarios. The coefficients ranged from 0.55 to 0.83 in the Italian sample and from 0.612 to 0.861 in the American sample.

We first checked the internal validity of the scenarios by performing three mixed ANOVAs, one for each control scale (happiness, responsibility, and reality), with the factor Scenario (Shame, Guilt, Embarrassment-1, embarrassment-2) and Nationality (Italian, American).

In the *happiness scale*, we found a significant interaction between Scenario and Nationality (F 3,483 = 258.5, *p* < 0.001, ηp2 = 0.62). In both groups, participants had a lower rating in the Shame (Italian, M = −3.85, SD = 1; American, M = −4.04, SD = 1.09) and Guilt (Italian, M = −4.05, SD = 0.89; American, M = −4.04, SD = 1.04), as compared to the Embarrassment-1 (Italian, M = −2.51, SD = 1.25; American, M = −3.42, SD = 1.3; *p* < 0.001) and Embarrassment-2 scenarios (Italian, M = −1.29, SD = 1.34; American, M = −2.76, SD = 1.62; *p* < 0.001). Furthermore, happiness ratings were significantly lower in Embarrassment 1 than in Scenario of Embarrassment-2 (*p* < 0.001).

Looking at the comparisons within the scenario across nationalities, we found that Italians had a higher rating for both scenarios, Embarrassment-1 and Embarrassment-2, compared to Americans (Embarrassment-1-Italian vs. Embarrassment-1-American: t_161_ = 4.47, *p* < 0.001; Embarrassment-2-Italian vs. Embarrassment-2-American: t_161_ = 6.25, *p* < 0.001, Bonferroni corrected). No difference was observed across groups in the other two scenarios.

In general, as expected, in each of the four conditions, participants perceived all the scenarios as reporting unhappy situations, confirming our experimental manipulation’s validity (Italian, M = −3.86, SD =1.00; American, M = −3.57, SD =1.38).

In the *responsibility scale*, we found a significant interaction between Scenario and Nationality (F_3,483_ = 427.6, *p* < 0.001, ηp^2^ = 0.73). As expected, in both groups, the perceived responsibility was higher in the Guilt scenario (Italian: M = 9.24; SD = 0.73; American: M = 8.26; SD = 2.01) compared to the other scenarios (Shame: Italian, M = M = 7.45; SD = 1.48; American, M = 6.25; SD = 1.74; Embarrassment-1: Italian, M = 4.29; SD = 2.04; American, M = 4.68; SD = 2.07; Embarrassment-2: Italian, M = 3.19; SD = 1.7; American, M = 3.48; SD = 2; *p* < 0.001). Also, we found that Italians had a higher rating for both Shame and Guilt scenarios compared to Americans (Shame-Italian vs. Shame-American: t_161_ = 4.72, *p* < 0.001; Guilt-Italian vs. Guilt-American: t_161_ = 4.39, *p* < 0.001). No difference was observed across groups in the other two scenarios.

In the *reality scale*, the ANOVA highlighted a significant interaction (F_3,483_ = 3.01, *p* = 0.03, ηp^2^ = 0.02). Specifically, we found that Italians had a higher rate in the Guilt scenario compared to Americans (Italian, M = 8.53, SD = 1.26; American: Guilt: M = 7.67; SD = 1.7; *t*_161_ = 3.89, *p* = 0.004). No difference was observed in the other three scenarios across groups. However, for both samples in each scenario, the average rating was higher than 7.5; thus, participants perceived the scenarios as realistic.

#### Emotion ratings within scenarios

**Figure 2:**
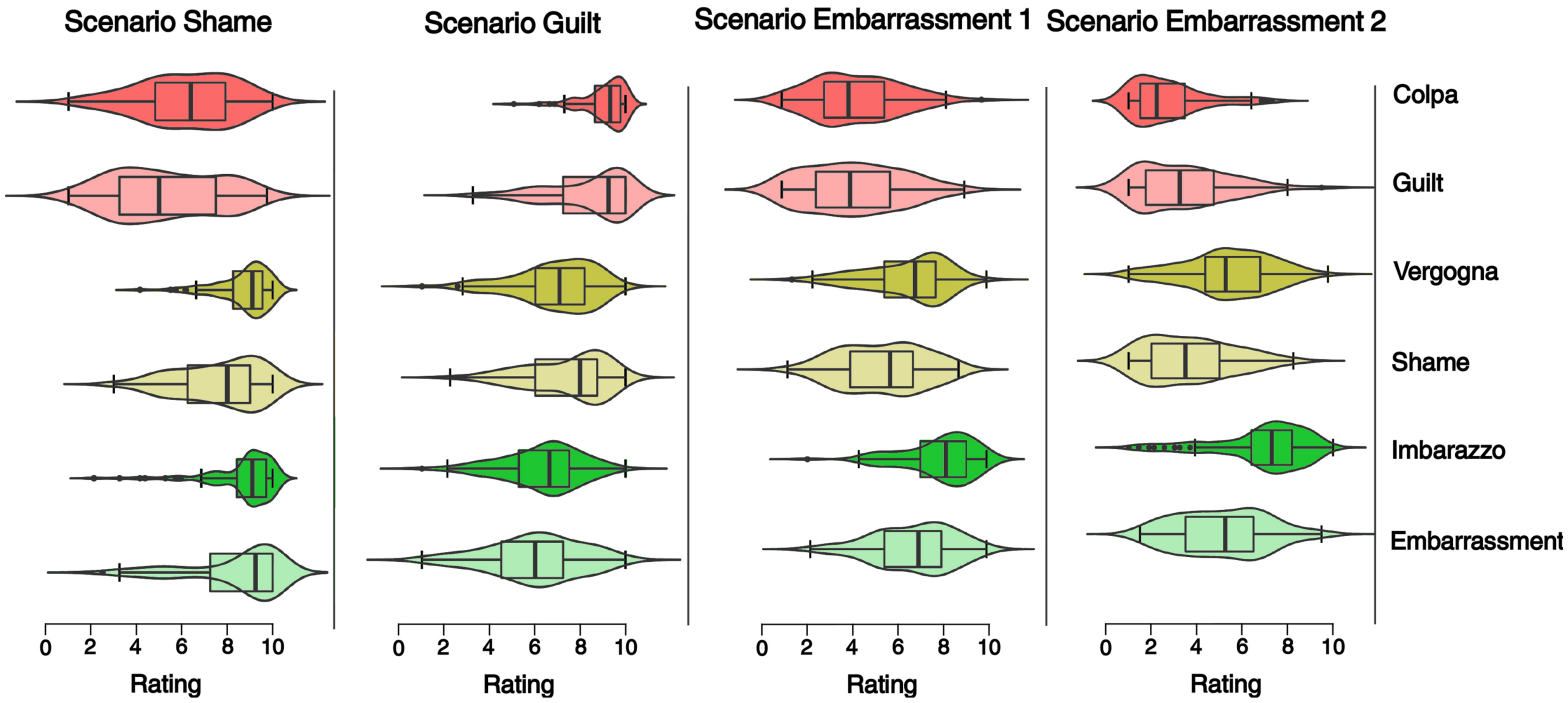
Emotions rating across scenarios in the Italian and American sample. The figure shows the violin plots with the superimposed box plots of the self-reported emotions intensity across the four scenarios (shame, guilt, embarrassment-1, embarrassment-2) for the emotions colpa/guilt, vergogna/shame, and imbarazzo/embarrassment. The violin plots show the distribution of ratings for each scenario. The box plots shows the median rating (the dark horizontal lines), the 75th percentile to the 25th percentile (the boxes), and the upper and lower boundaries 1.5*75th (or 25th) quartiles (the whiskers).

What is the emotion label with the highest rating in each scenario type? In this respect, is there any difference between Italians and Americans? To answer these questions, we computed four mixed ANOVAs, one for each scenario type, with the conditions Emotion (guilt/colpa, shame/vergogna, embarrassment/imbarazzo; within-subjects factor) and Nationality (Italian, American; between-subjects factor; Figure 2).

In the *Guilt scenario*, the results revealed a significant interaction between Emotion and Nationality (F_14.92,1.55_ = 9.62, *p* < 0.001, ηp^2^ = 0.056). In particular, post-hoc comparisons showed that guilt/colpa were the emotion labels that received the highest rating in both groups, followed by shame/vergogna (American, Italian) and then embarrassment/imbarazzo (American, Italian, *p* < 0.001). Moreover, guilt/colpa was higher in Italians compared to Americans (*t*_161_ = 3.147, *p* = 0.029).

In the *Shame scenario*, the results showed a significant main effect of Emotion (F_1.37,220.58_ = 534.39, *p* < 0.001, ηp^2^ = 0.52) and Nationality (F_1,161_ = 12.1, *p* < 0.001, ηp^2^ = 0.07). *Post-hoc* comparisons showed that Italians gave overall higher ratings than Americans (*t*_161_ = 3.48, *p* < 0.001). Notably, the emotion label which received the highest rating was embarrassment/imbarazzo (embarrassment/imbarazzo vs. guilt/colpa: *t*_161_ = −13.99, *p* < 0.001; shame/vergogna vs. embarrassment/imbarazzo: *t*_161_ = −3.50, *p* = 0.002). The second highest emotion was shame/vergogna which received a higher rating than guilt/colpa (*t*_161_ = −13.95, *p* < 0.001).

**Figure 3:**
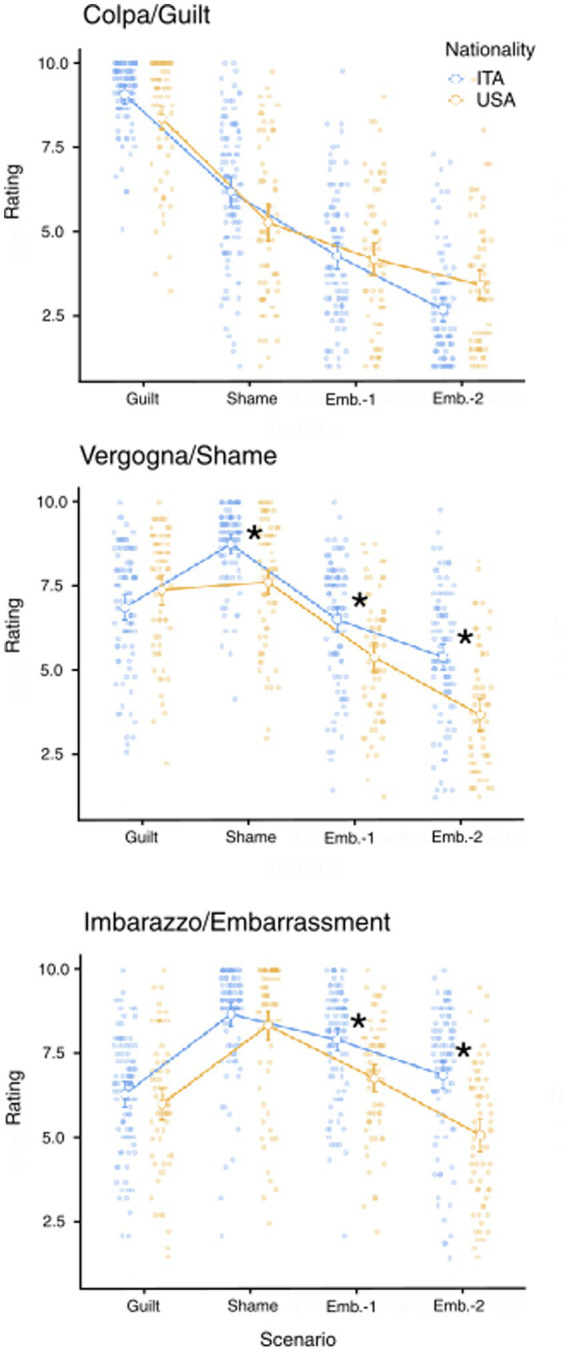
Emotions rating in the three target emotions in the Italian and American sample. Observed ratings and estimated marginal means for guilt/colpa, shame/vergogna, and embarrassment/imbarazzo. The lines are for display purpose only. *p < 0.05 Bonferroni multiple comparison correction.

Since the interaction was not significant, we computed two separate ANOVAs, one for each group with the factor Emotions. In the Italian group, the results showed a significant main effect of Emotions (F_5,485_ = 156.11, *p* < 0.001, ηp^2^ = 0.60). Post-hoc comparisons showed that participants rated most highly the emotions vergogna and imbarazzo compared to colpa (colpa vs. vergogna: t_97_ = −12.71, *p* < 0.001; colpa vs. imbarazzo: t_97_ = −12.64, *p* < 0.001). No difference was observed between vergogna and imbarazzo (t_97_ = 0.655, *p* = 1). In the American group, we found a significant main effect of Emotions (F_5,320_ = 51.4, *p* < 0.001, ηp^2^ = 0.45). Post-hoc comparisons showed that participants rated higher the emotion term embarrassment compared to shame (t_64_ = −4.31, *p* < 0.001) and guilt (t_64_ = −7.92, *p* < 0.001). In addition, shame received a higher rating than guilt (t_64_ = −7.63, *p* < 0.001).

In the *Embarassment-1 scenario*, the results showed a significant interaction between Emotions and Nationality (F_14.92,1.55_ = 9.55, *p* < 0.001, ηp^2^ = 0.056). Post-hoc comparisons showed that Italians rated both imbarazzo and vergogna higher than Americans (embarrassment and shame; embarrassment: *t*_161_ = 4.315, *p* < 0.001; shame: *t*_161_ = 3.811, *p* < 0.003). As expected, in both groups, embarrassment/imbarazzo was the highest emotion (Italian, M = 7.94, SD = 1.58; American, M = 6.79, SD = 1.76), and shame/vergogna was the second one (Italian, M = 6.51, SD = 1.81; American, M = 5.4, SD = 1.86, *p* < 0.001).

In the *Embarrassment-2 scenario*, overall, we found the same pattern observed in the Embarrassment-1 scenario. Again, we found a significant interaction between Emotions and Nationality (F_14.92,1.55_ = 90.77, *p* < 0.001, ηp^2^ = 0.242), and post-hoc comparisons showed that Italians rated both imbarazzo and vergogna higher than Americans (embarrassment: *t*_161_ = 5.50, *p* < 0.001; shame: *t*_161_ = 5.56, *p* < 0.003). Moreover, embarrassment/imbarazzo was the highest emotion (Italian, M = 6.88, SD = 2.04; American, M = 5.11, SD = 1.98), and shame/vergogna was the second one (Italian, M = 5.40, SD = 1.94; American, M = 3.68, SD = 1.91, *p* < 0.001).

#### Emotion ratings between scenarios

What is the scenario with the highest rating for each emotion label? Is there any difference between Italians and Americans? To address these questions, we computed three mixed ANOVAs, one for each emotion label, with the conditions Scenario (Guilt, Shame, Embarrassment-1, Embarrassment-2; within-subjects factor) and Nationality (Italian, American; between-subjects factor; Figure 3).

Regarding the emotion terms guilt/colpa, the results showed a significant interaction between Scenario and Nationality (F_64.2,2.55_ = 9.21, *p* < 0.001, ηp^2^ = 0.055). Specifically, we found the same pattern of ratings across groups: the Guilt scenario was associated with the highest rating of guilt/colpa, followed by shame/vergogna, and ultimately embarrassment/imbarazzo (lowest *p* < 0.001). Moreover, looking at the comparisons within the scenario, we found no differences across groups.

In the emotion shame/vergogna, we found a significant interaction between Scenario and Nationality (F_64.2,2.55_ = 18.1, *p* < 0.001, ηp^2^ = 0.10). The comparisons within the scenario showed that Italians rated this emotion higher than Americans in all the scenarios except for Guilt (highest *p* = 0.006, lowest *p* < 0.001). Interestingly, while in the Italian sample, the emotion shame/vergogna was higher in the Shame compared to the Guilt scenario (*p* < 0.001), in the American sample, there was no difference between the two scenarios (*p* = 1).

In the emotion embarrassment/imbarazzo, the interaction between Scenario and Nationality was significant (F_64.2,2.55_ = 10.3, *p* < 0.001, ηp^2^ = 0.06). As for the emotions shame/vergogna, we found that Italians rated embarrassment/imbarazzo higher than Americans in all the scenarios except the Guilt (highest *p* = 0.048, lowest *p* < 0.001). We also found that in both groups, these emotions received the highest rating in the Shame, followed by the Embarrassment-1 scenario (highest *p* = 0.036, lowest *p* < 0.001).

#### Shame vs. vergogna

We hypothesized that the Anglo-Saxon word shame would contain a moral connotation not associated with the Italian word vergogna. To address this question, we looked at the response distributions of this emotion in the Shame and Guilt scenarios and computed a series of chi-squared tests. Specifically, for each scenario (Shame and Guilt) and sample (Italian and American), we created a distribution of frequencies that comprised: frequency of coherent rating, incoherent rating, and equal rating. For the “coherent rating,” we counted how many times individuals gave the highest rating to the emotion coherent with the scenario (e.g., emotion “shame/vergogna,” scenario “Shame”). For the “incoherent rating,” we counted how many times individuals gave the highest rate to the emotion incoherent with the scenario (e.g., emotion “shame/vergogna,” scenario “Guilt”). Ultimately, for the “equal rating,” we counted how many times individuals gave the same rating to the two emotions.

We first compare the two scenarios in both samples. We found a significant difference in the rating distribution only in the American sample (Italians: χ_22,784_ = 1.24, *p* = 0.537; Americans: χ_22,784_ = 34.1, *p* < 0.001), where the difference between the coherent and incoherent ratings was higher in the Shame compared to the Guilt scenario. This difference implies that in the American group the emotion shame in the Guilt scenario received more often a higher rating than the emotion guilt in the Shame scenario.

Next, we compared the two groups across scenarios and found that they significantly differed in frequency distributions (χ_22,1,281_ = 12.06, *p* = 0.002). Notably, Americans seemed to have higher levels of shame in the Guilt scenario compared to Italians.

#### Correlations between guilt, shame, and embarrassment

We assessed the degree of relationship between the target emotions with a series of partial correlations performed independently in the two groups. For each correlation, we controlled for the validity scales (happiness, responsibility, and reality) and the other emotions (fear, anger, and regret).

In the Italian sample we found a positive correlation between “vergogna” and “imbarazzo” in all scenarios (Shame, *r* = 0.599, *p* < 0.001; Guilt, *r* = 0.675, *p* < 0.001; Embarrassment-1, *r* = 0.696, *p* < 0.001; Embarrassment-2, *r* = 0.738, *p* < 0.001), and “colpa” and “imbarazzo” in the Shame scenario (*r* = 0.311, *p* = 0.003).

In the American sample, we found a partially different pattern of correlations. In particular, we found a positive correlation between shame and embarrassment in the Shame (*r* = 0.704, *p* < 0.001) and Guilt (*r* = 0.569, *p* < 0.001) scenarios. In the Embarrassment-1 and-2 scenarios, we found a positive correlation between shame and guilt (Embarrassment-1: *r* = 0.440, *p* < 0.001; Embarrassment-2: 0.466, *p* < 0.001). Moreover, in Embarrassment-1, we also found a positive correlation between shame and embarrassment (*r* = 0.527, *p* < 0.001) and between embarrassment and guilt (*r* = 0.292, *p* = 0.025).

## Discussion

We all experience many emotions in everyday life, often more than one simultaneously. This human ability could make it difficult to distinguish which emotion we are feeling at a specific time and the event that affected such emotion. Moreover, this process becomes more complex if our emotions share some ingredients, such as shame, guilt, and embarrassment.

It is difficult to elicit a “pure” moral emotion because different emotions often co-occur (Izard, 1991). Following a social transgression, for example, individuals may feel guilty about their wrongdoing while at the same time feeling embarrassed/ashamed because the event was witnessed by others (Finger et al., 2006). Even though they share many features, several authors pointed out that they are different emotions by highlighting their differences. However, in the literature, there is no consensus yet regarding these distinctive features and how they are modulated by cultural factors. Thus, the starting point of this research was the paucity of studies comparing these three emotions, especially in a cross-cultural context and, specifically, the lack of research comparing the Italian and American cultures (Bastin et al., 2016; Higgs et al., 2020).

Our study aimed to address two main questions. First, we aimed to test whether the elements characterizing the three emotions, as theorized by Miceli and Castelfranchi (2018), Castelfranchi (1998), and Castelfranchi and Poggi (1990) reflected the emergence of the three target emotions. Second, we sought to compare emotions ratings in two different cultures, the Italian and the American. We hypothesized that the major difference between the two cultures would be related to the two emotion terms indexed by shame and vergogna.

To answer these questions, we built 16 scenarios divided into four groups to induce the target emotions. Overall, we found that Guilt and Embarrassment scenarios were effective in eliciting primarily the target emotions, guilt/colpa, and embarrassment/imbarazzo, respectively. Conversely, with the Shame scenario, we found mixed results as embarrassment/imbarazzo was the emotion that received the highest rating. However, shame/vergogna received the highest rating in the Shame scenario compared to the others. Moreover, we found that shame was equally elicited only in the American sample by the Shame and Guilt Scenarios. This result was further supported by the distributions of the response frequencies, where we found that Americans rated shame as the highest secondary emotion in the Guilt scenario differently than Italians.

Furthermore, we found important differences between the two groups in how the emotions ratings were associated. Specifically, we found that in the Italian group, vergogna and imbarazzo were positively associated in all the scenarios. Conversely, the American group showed a more complex pattern of correlations. Indeed, we found that shame and embarrassment were positively correlated in all the scenarios except for Embarrassment-2; shame and guilt were positively correlated in Embarrassment-1 and -2; and guilt and embarrassment were positively associated in Embarrassment-2.

Regarding the emotion labels guilt/colpa and embarrassment/imbarazzo we observed a convergence of results between Italians and Americans, as in both groups, these emotions received the highest rating in the Guilt and Embarrassment scenarios, respectively. Moreover, there was no difference in how subjects rated the emotion terms guilt/colpa across scenarios. These results suggest that the elements used to build Guilt and Embarrassment scenarios were effective in primarily evoking these emotions in both groups. This pattern is consistent with a study that found that guilt, as defined by British and American English and Polish cultures, is less varied cross-linguistically than shame, and that embarrassment is the most coherent lexical concept/category across the three linguistic communities, and it has a single relatively stable profile (Krawczak, 2014).

Although, in the Shame scenario embarrassment/imbarazzo were the emotion terms that received the highest ratings, followed by shame/vergogna and then guilt/colpa, the terms shame/vergogna received the highest rate in the Shame scenario compared to the Guilt and Embarrassment scenarios. These results confirm the validity of the elements used to build the scenarios and suggest that shame/vergogna and embarrassment/imbarazzo are closely related. Indeed, in both groups, these two emotional terms were positively correlated in almost all scenarios, as if both emotion terms shared a similar mental state. Moreover, this pattern of results points to some overlap between these emotion categories, especially with the scenario Shame which involves a situation in which the self-image is compromised and the tendency is to look down and disappear. Indeed, the two terms are often used interchangeably in common parlance.

Some authors (e.g., Goffman, 1967; Zimbardo, 1977) group these emotion terms in a single category, while other authors locate them at different points on a two-dimensional representation whose dimensions refer, respectively, to the seriousness of the transgression and the extent of one’s fault, for it stressing the moral dimension of embarrassment (Harre, 1983); still, others distinguish them in terms of severity of inadequacies (Buss, 1980); alternatively, embarrassment is linked to etiquette, and shame to moral worth (Schlenker and Leary, 1982); other authors distinguish them for several characteristics (Castelfranchi and Poggi, 1990; Tangney et al., 1996). These last observations have been supported by studies that show that participants remember experiences of embarrassment, in comparison to shame, as caused by accidents, as less related to moral standards, as less severe, of shorter duration, and occurring more often only because others knew ‘about it’ (Miller and Tangney, 1994; Tangney et al., 1996; Sabini et al., 2001).

It has been suggested that the link between the two emotions is quite strong: not knowing what to do because of embarrassment may subject one to make a negative evaluation and hence cause shame; on the other hand, shame may prevent one from knowing what to do. In such situations, it would not be easy for a subject to report whether blushing was caused by shame or embarrassment. Castelfranchi and Poggi (1990) theorized that the two emotions terms can often correlate with each other in that shame aims to defend the individual’s image. In contrast, the function of embarrassment is to avoid intrusion into the private sphere of others. Nevertheless, these two emotions can be interconnected in complex ways, as emerges from the results of our study. It has also been shown that attempts to differentiate the two emotions based on characteristics are not always confirmed by experimental data and daily life situations (Carnì et al., 2013). There may be various reasons for this difficulty; for example, in real life, the two emotions are often co-present, and the instruments used to evaluate emotions are often constructed according to a given model that conceptualizes each emotion rather rigidly. Moreover, some have also pointed out the difficulty in defining some emotions in a way acceptable to various researchers and authors (Carnì et al., 2013).

Interestingly, we also found that the Italian sample gave higher scores on the happiness scale in scenarios of Embarrassment 1 and 2 compared to the American sample. Therefore, it seems that imbarazzo is less painful than embarrassment and, therefore, more distant from vergogna, and that the distance between imbarazzo and vergogna is shorter than the distance between embarrassment and shame. Castelfranchi and Poggi (1990) already hypothesized this difference in the labels between the Italian and the Anglo-Saxon culture as if the terms that referred to moral emotions in the two cultures did not label the same emotional and physiological experience. According to the authors, the semantic areas linked to the words for embarrassment and shame in English and Italian only partially overlap. The English word embarrassment covers at least some part of the meaning that in Italian is linked to vergogna. In particular, the term embarrassment seems to include the idea of some shortcoming of the individual, some inadequate feature or behavior, that in Italian is implied by the word vergogna, but not necessarily by the word imbarazzo.

From a neuroanatomical point of view, the two emotions share functional cortical substrates, including the hippocampal and mid-brain regions (Bastin et al., 2016). It has been argued that the role of the hippocampus in responding to psychosocial stress (McEwen, 2001; Dranovsky and Hen, 2006) could arise from the association of both emotions with the threat from the external environment toward the self as theorized by Tangney et al. (1992).

Importantly, we found that shame was equally elicited only in the American group by the Shame and Guilt scenarios, and this term was rated more often as the second highest emotion in the Guilt scenario, compared to Italians. Moreover, shame and guilt were positively correlated in the American group in all Embarrassment scenarios. These findings support the hypothesis that shame is closer to guilt for Americans, consistent with individualistic cultures being guided by internal moral standards rather than external norms and expectations. These results are in line with a psycholinguistic study that found that in the Anglo-Saxon communities, compared to Poland, the sources of shame are linked to internally defined moral standards and are closer to guilt (Krawczak, 2014). Unlike shame, vergogna has been referred not only to moral flaws but also to simple clumsiness. Conversely, shame, with its stress on moral matters, looks closer to guilt, which seems to share a semantic element of responsibility (Silver et al., 1987). This is one further divergence of shame from vergogna, which may refer to physical defects or even to one’s good luck, for which one is not responsible.

In line with our findings, Hurtado de Mendoza et al. (2010) suggested that the cluster of constitutive elements of the concept “verguenza,” used as a Spanish translation of shame, can be quite different from the constitutive features of shame. In this study, the Authors compared American and Spanish students about several characteristics of the two emotions. The American students, as already highlighted by several studies in the literature, described shame as an unpleasant feeling caused by negative social evaluations (Lewis, 2000; Tangney and Dearing, 2002; Tracy et al., 2009) related to internal attributions and morality (Niedenthal et al., 1994; Wallbott and Scherer, 1995; Greenwald and Harder, 1998; Rodrıguez Mosquera et al., 2000; Smith et al., 2002; Scheff, 2003; Baldwin and Baccus, 2004; Gilbert, 2004; Leary, 2004; Parrott, 2004) and the incongruence between ego real and ego ideal (Higgins, 1987; Lazarus, 1993; Demos, 1996; Tracy et al., 2009) so appearing closer to guilt than to embarrassment (Tangney and Fisher, 1995). In contrast, most features of verguenza mentioned by Spanish students were related to external evaluations rather than internal attributions and morality, and liked to situations in which the person feels socially uncomfortable. These features highlight the connection between “verguenza” and the concept of shyness and embarrassment (Mosher and White, 1981; Abu-Lughod, 1990; Scheff, 2003; Baldwin and Baccus, 2004; Fessler, 2004; Li et al., 2004). Indeed, the only English category that did not differ significantly from “verguenza” was embarrassment. Of course, some features were rated as very typical for “verguenza” and shame (unpleasant feeling, feeling uncomfortable, the feeling caused by your or others’ behavior, disappearing or feeling imposed by society), which suggests that the concept of shame includes features related to morality as well as features related to reputation and social evaluation (Castelfranchi and Poggi, 1990; Miceli and Castelfranchi, 2018). Within the English categories, shame and guilt are closer to each other than shame and embarrassment (Hurtado de Mendoza et al., 2010), contrary to what happens in our study in the Italian sample. Their results suggest that verguenza and shame should not be considered equivalent categories because they differ in the degree of typicality of their features and their affective meaning. According to the authors, the close affective meaning of verguenza and embarrassment should not lead to the easy conclusion that verguenza should be translated as embarrassment because these findings are not informative about the content and internal structure of those two categories. Conversely, they highlighted that the categories of emotional experience corresponding to shame and verguenza encompass non-overlapping features and significantly differ in the typicality ratings of 25 out of 29 features. These differences could represent a different connotation of the two emotions in an Anglo-Saxon and a Latin country.

From a neuroanatomical view, shame and guilt (but not embarrassment) appeared to be associated with anterior insular cortex activity connected to emotional and cognitive aspects of pain (Wiech et al., 2014; Pavuluri and May, 2015). This result confirms the idea of shame and guilt as more painful and damaging negative emotions than embarrassment (Tangney et al., 2005). Shame and guilt were also both associated with dorsal anterior cingulate cortex function that has been correlated with the experience of negative affect (Mayberg et al., 1999) and the experience of social pain (Eisenberger and Lieberman, 2004; Masten et al., 2011). Several brain regions appeared to be ‘guilt-specific’, including the ventral anterior cingulate cortex, which has been associated with the inhibition of emotions (particularly fear), and it has been suggested that its activation may be associated with emotion regulation by facilitating the planning of adaptive response (Etkin et al., 2011).

Finally, Italians score higher than Americans in most emotion ratings in all scenarios. This result could derive from a greater perception or expression of these emotions in the Italian sample. An interesting datum in the literature is that the Spanish people seem to experience a greater intensity of emotions than the Americans, as reported by Hurtado de Mendoza et al. (2010). Therefore, this pattern might be related to possible differences across individualistic and collectivistic cultures in experiencing or expressing emotions. However, we do not have enough elements to distinguish which of the two hypotheses is plausible or if there is a co-occurrence of both elements. Further studies in this direction are necessary to deem between the two hypotheses.

As described in detail elsewhere (Berry et al., 1992), it is essential to highlight that what may appear to be cross-cultural differences in emotion ratings may be due to response biases or a lack of construct validity across populations. Many researchers put aside their knowledge of these difficulties and either rely on dictionaries (Hupka et al., 1999) or apply cursory back-translations (Matsumoto and Ekman, 1989; Mauro et al., 1992; Scherer and Wallbott, 1994; Rozin et al., 1999; Eid and Diener, 2001; Mondillon et al., 2005). As such, it is necessary to ensure psychometric equivalence before making meaningful comparisons (Thielmann et al., 2020). Hurtado de Mendoza et al. (2010), as previously described, demonstrated that the constitutive features of the usual Spanish translation of shame could be quite different from the constitutive features of the shame itself. There is much empirical evidence of significant cultural differences in the categorization of the emotional experience, suggesting that the translations of shame do not always activate the same cluster of conceptual features (Wierzbicka, 1986; Abu-Lughod, 1990; Parish, 1991; Menon and Shweder, 1994; Iglesias, 1996; Hurtado de Mendoza and Parrott, 2002; Fessler, 2004; Pascual et al., 2007). All these data contribute to an increase in the perceived need to find an alternative way to use verbal labels referring to emotions in studies comparing different cultures (Hurtado de Mendoza et al., 2010). Indeed, the one-to-one translations of the labels into other languages, especially for shame in this case, is problematic and highlights the necessity of searching for alternative ways to the encyclopedic method.

Comparing our three target labels, it seems that the semantic domains related to the words for guilt, shame, and embarrassment in English and Italian do not completely overlap. Indeed, the English word shame seems to cover at least part of the meaning that vergogna carries in Italian and it usually translates with shame, disgrace, and embarrassment. On the other hand, the English word embarrassment seems to include the idea of an individual’s flaws, characteristics or inadequate behavior, which in Italian is implied by the word vergogna, but not necessarily with the word imbarazzo. The minimal necessary core meaning of the Italian word imbarazzo does not even mention an emotion; it can simply mean a conflict between different options: not knowing what to do or choose because all choices are equally bad or good. This word, therefore, covers a more limited scope than the English word embarrassment because it does not necessarily imply any shortcomings of the embarrassed person. This semantic domain is covered by vergogna. So, by comparison, embarrassment seems to extend to vergogna territory. Unlike shame, vergogna can refer not only to moral shortcomings but also to simple clumsiness. In this case, shame, with its emphasis on moral issues, is similar to “senso di colpa,” which seems to share a semantic element of responsibility (Silver et al., 1987). Finally, from a linguistic standpoint, “guilt” and its Italian equivalent, “senso di colpa” seem to overlap more than the labels for embarrassment and shame in that both terms seem to share important elements related to feeling responsible for unjust harm caused to others.

Importantly, when people are asked to label or rate an event or an emotion, they refer to the first association that comes to mind. This process might be affected by the absence of all the elements and variables that emerge when a person is directly involved in an event or experiencing an emotion in a real situation (Zammuner, 1995). Moreover, D’Errico and Poggi (2014) highlighted how traditional survey methods studying emotions, such as self-report questionnaires, can be improved by modifying the analysis methodology by adding, for example, automatic lexicographic analysis. This analysis would better characterize similarities and differences in the lexicon connected to different emotions.

Overall, participants perceived the scenarios as realistic, rating them as unhappy, confirming our experimental manipulation’s validity. Moreover, as expected, they perceived responsibility rating higher in the Guilt scenario than the others. This result confirms that the elements theorized by Miceli and Castelfranchi (2018) and Miceli and Castelfranchi (1998) are constitutive of each specific emotion at a cognitive, physiological, and situational level, indicating a correspondence between the experience of the emotion and the label attributed. The correspondence would facilitate the study of emotions and understanding others’ experiences in clinical practice.

## Constraints on generality and conclusion

The stimuli consisted in written scenarios based on the elements suggested by Castelfranchi and colleagues. Thus, we except the results to generalize to situations in which participants rate similar scenarios, as long as the scenarios meet the defining criteria proposed by the authors. Moreover, pilot studies from our lab resulted in similar results despite variations in the web platforms used to administered the experiments. Therefore, we do not expect such variations to be significant. We think that the results will be reproducible with Italian and American individuals from similar subject pool serving as participants. We have no reason to believe that the results depend on other characteristics of the participants, materials, or context.

In this study, we aimed to investigate emotions ratings by using scenarios where the different prototypical elements were intertwined. Thus, a possible limitation of this study was that we were not able to differentiate the specific and direct impact of each element on subjects’ ratings. Future studies should take into consideration, through subjects’ ratings, the extent to which each of the scenarios contains each of the elements and use the strength of elements as predictors of the emotions ratings.

We used only one exclusion criterion for the enrollment of the subjects, namely the presence of alexithymia. Therefore, other relevant variables, such as the intake of psychotropic drugs, the presence of psychiatric and neurological disorders, the presence of cognitive deterioration, and the presence of alcohol and drug addiction, were not controlled. Moreover, we did not record participants’ linguistic skills, educational qualifications, and profession, which represent important factors in cross-linguistic studies to be taken into consideration.

Furthermore, the results were discussed in the context of differences between individualistic Vs. collectivistic cultures; however, we did not measure how this dimension varied in our samples. Thus, future cross-cultural studies should directly measure the individualism–collectivism variable through appropriate instruments, such as the 13 statements developed by Sinha and Verma (1994). The authors coined the term coexistence to describe a model that allows many elements, including contradictory ones, to coexist within a culture and a person. The mode of coexistence separates the private self from the public one. The public self is dominated by collectivist values, such as family loyalty, intra-group solidarity, and national identity; it coexists with the private self that maintains personal values of personal cultivation and endeavors. Thus, this scale would allow to measure how this dimension covariates with the self-reported emotions ratings.

Lastly, it is also important to note the differences concerning cultural contexts that could influence the emotional response on a topic like responsibilities, duty, and social expectations. For instance, collectivist cultures seem to associate interpersonal responsibilities with family and friends, while individualistic cultures, like European and American, consider similar responsibilities in obligatory terms (Miller et al., 2008). Even though Italian and American cultures share some individualistic aspects, their cultural differences should be considered since they could influence the emotional experience of events. Moreover, apart from cultural contexts and differences, another important factor that should be considered is who is the subject of evaluation or judgment inside this context. Indeed, people seem more prone to experience good emotions toward a third person perceived as the victim, while the contrary happens when we judge ourselves. So, in rating emotions after reading a story, possible differences could be accounted for by the different individuals’ perspectives, especially when asking to evaluate social emotions, such as guilt, shame, and embarrassment (Malti and Keller, 2010).

In conclusion, further studies are needed to deepen the results that emerged in our study with a more controlled sample and appropriate scales that take into account the relevant cultural variables and by manipulating the different ingredients used to evoke the perceived emotions.

## Data availability statement

The datasets presented in this study can be found in online repositories. The names of the repository/repositories and accession number(s) can befound at: the Open Science Framework (OFS) and can be accessed at https://osf.io/txdyp/.

## Ethics statement

The studies involving humans were approved by Ethics Committee of the Association of Cognitive Psychotherapy (APC-SPC; Prot. N. 8-2023). The studies were conducted in accordance with the local legislation and institutional requirements. The participants provided their written informed consent to participate in this study.

## Author contributions

CG: Data curation, Formal analysis, Investigation, Methodology, Visualization, Writing – original draft, Writing – review & editing, Conceptualization, Supervision, Validation. FS: Data curation, Formal analysis, Investigation, Methodology, Visualization, Writing – original draft, Writing – review & editing. AC: Data curation, Formal analysis, Investigation, Methodology, Visualization, Writing – original draft, Writing – review & editing. EE: Data curation, Formal analysis, Investigation, Methodology, Visualization, Writing – original draft, Writing – review & editing. AM: Data curation, Formal analysis, Investigation, Methodology, Visualization, Writing – original draft, Writing – review & editing. CC: Conceptualization, Supervision, Validation, Writing – review & editing. FM: Conceptualization, Funding acquisition, Investigation, Methodology, Resources, Supervision, Validation, Writing – review & editing.

## Footnotes

^1^ https://osf.io/txdyp/

^2^ https://www.google.it/intl/it/forms/about/

^3^ https://www.testable.org/

^4^ https://www.jamovi.org
