# Supplementary material for: Guilt, shame, and embarrassment: similar or different emotions? A comparison between Italians and Americans
Source: Front Psychol. 2023 Dec 12;14:1260396. doi: 10.3389/fpsyg.2023.1260396 (PMC10773588; doi:10.3389/fpsyg.2023.1260396)
Supplement: Supplementary file 1 [file Data_Sheet_1.PDF]

Supplementary Information

Examples of scenarios used in the experiment for each emotion label.

Guilt

Marco invites the investors in his project out for dinner. He chooses the restaurant, whose specialty is fish, and promises everyone that the food is very good. However, the next day at work, he learns that half of his guests felt ill because of the dinner, with strong gastrointestinal symptoms. Since fishing had been suspended in that period, there was probably some problem with frozen fish and his guests reported symptoms similar to those associated with food poisoning. “I chose the restaurant and vouched for its quality. I could have been more careful! They are such nice and kind people. They deserved more care on my part. Now how can I make it up to them? How can I make them understand that I’m really sorry and that I’d like to help them?” Marco feels a strong sense of tightening in his chest and can’t stop feeling bad about the choice he made and its outcome.

Shame

Francesco has a slight physical malformation of his right arm and shoulder, which partially limits its functioning. He is a nurse and this is his first day of work in his new position. He has to empty the bedpan of a bedridden patient. While picking it up, his right arm locks and the bedpan falls on the floor, emptying out its contents, in front of his new supervisor. Francesco turns red in the face and wants to disappear. He starts to think: Look at what I’ve done, I don’t even know how to empty a bedpan! Now my supervisor will think that I’m not capable of doing this job given that I can’t even manage to do something so simple . . .!

Embarrassment-1

Mario is at a party with some friends. They’re at a discotheque. While he is in line at the bar to order a cocktail, he sees his best friend’s girlfriend walk by, hand in hand with another boy. Suddenly she turns around and sees him. Mario doesn’t know what to do. He doesn’t know if he should say something to her but risk seeming like an intrusive gossip or to pretend not to have seen her, even though doing that he would risk looking like someone who doesn’t care about his friend.

Embarrassment-2

Emanuele is at the supermarket. In front of the milk refrigerator there is a couple that is having a spirited argument. Emanuele has already selected everything that he needs except for some milk and he has no way of getting it without disturbing the couple. Emanuele starts to think: What di I do? Do I interrupt them to tell them to step aside or do I stay behind them and let them notice me by themselves. If I stand behind them

without saying anything they might think that I'm eavesdropping on them. But I really need the milk. I don’t know what’s the best thing to do. What a situation!
